# Modeling the HIV-1 Intasome: A Prototype View of the Target of Integrase Inhibitors

**DOI:** 10.3390/v2122777

**Published:** 2010-12-21

**Authors:** Zhiqi Yin, Robert Craigie

**Affiliations:** Laboratory of Molecular Biology, National Institute of Diabetes and Digestive and Kidney Diseases, National Institutes of Health, Bethesda, MD 20892, USA

**Keywords:** HIV-1, integrase, intasome

## Abstract

The HIV-1 integrase enzyme is essential for integrating the viral DNA into the host chromosome. Infection is aborted in the absence of integration, making integrase an attractive antiviral target. Recently approved inhibitors of integrase bind tightly to integrase assembled in a nucleoprotein complex with the viral DNA ends (intasome), but have only low affinity for free integrase. High-resolution structures of HIV-1 intasomes are therefore required to understand the detailed mechanisms of inhibition and resistance. Although the structure of the HIV-1 intasome has not yet been determined, the structure of the related prototype foamy virus (PFV) intasome was recently solved. A new study [1] exploits the PFV structure to model the HIV-1 intasome. The model provides the most reliable picture to date of the active site region of the HIV-1 intasome and is an important advance in studies of inhibition of this essential HIV-1 enzyme.

Integrase catalyzes the key DNA cutting and joining reactions responsible for integrating retroviral DNA into cellular DNA, where it is replicated along with host DNA during multiple cycles of cell division and serves as the template for transcription of progeny viral RNAs (reviewed in [2]). The viral DNA is synthesized by reverse transcription in the cytoplasm, after which it remains associated with other viral and cellular proteins as a large nucleoprotein complex called the preintegration complex (PIC). The PIC is later transported to the nucleus where integrase within the PIC splices the viral DNA into the host genome. Integrase normally functions as part of the PIC, but integrase alone is able to carry out the key DNA cutting and joining steps *in vitro* with simple DNA substrates that mimic the viral DNA ends in the presence of a divalent metal ion. In the first of these reactions, 3′ end processing, integrase removes two nucleotides from each 3′ end of the viral DNA. In the second step, DNA strand transfer, the 3′ hydroxyls of the terminal adenosine exposed by 3′ end processing attack a pair of phosphodiester bonds at the site of integration into target DNA. In the case of HIV-1, the sites of integration on the two target DNA strands are separated by five nucleotides resulting in a five base pair duplication of target DNA sequence flanking the integrated viral DNA after repair of the resulting integration intermediate by cellular enzymes. A key nucleoprotein intermediate is the intasome, comprised of a tetramer of integrase that stably bridges a pair of viral DNA ends [3]. Recently developed integrase strand transfer inhibitors (INSTI), such as Raltegravir and Elvitegravir, bind to the intasome and have only weak affinity for free integrase in the absence of bound viral DNA ends. High-resolution structures of the HIV-1 intasome are therefore required to understand the mechanism of inhibition and resistance, and to design improved drugs.

Structural studies of retroviral integrases have been fraught with obstacles and progress has been painfully slow. Although it is well over a decade since the structures of all three domains of HIV-1 integrase were determined [4–7], the first structure of a retroviral intasome—that of prototype foamy virus (PFV)—has only recently been solved [8]. The first problem was the poor solubility of intact HIV-1 integrase that was partially overcome by studying individual domains rather than the intact enzyme. A mutation was also introduced into the catalytic domain to improve its solubility and facilitate crystallization [9]. Later, two domain structures were determined for both the N-terminal domain and catalytic domain and for the C-terminal and catalytic domain (reviewed in [10]). Structures of different retroviral integrases exhibited major differences in the spatial configuration of the catalytic and C-terminal domain suggesting that bound viral DNA is required to constrain the active integrase multimer in the intasome. Numerous models of the intasome have been proposed based on these partial structures, but the PFV intasome structure demonstrated that all of them are wrong.

The breakthrough in structural studies of intasomes came from the discovery that PFV integrase displays much more favorable properties for biophysical studies than its HIV-1 counterpart [11]. It exhibits better solubility and efficiently catalyzes coupled integration of pairs of short oligonucleotides mimicking the viral DNA ends *in vitro*. In contrast, the products of *in vitro* reactions with HIV-1 integrase are mainly insertions of single viral DNA ends into one strand of target DNA, a reaction that lacks the full fidelity of integration *in vivo*. The ability of PFV integrase to support efficient coupled integration of pairs of viral DNA ends was critical to the success of the structural studies because intasomes only assemble under conditions that support coupled integration. Assembly of HIV-1 intasomes also requires much longer DNA that is unsuitable for crystallization. The crystal structure of the PFV intasome [8] provided the first view of any retroviral intasome. The structure could not have been predicted based on the structure of the HIV-1 integrase protein domains alone because the viral DNA acts as a glue that holds the intasome together with the integrase domains adopting a very different spatial arrangement compared with the earlier structures of the protein alone (Figure 1). An inner pair of integrase subunits within the tetramer in the PFV intasome is responsible for all the protein-protein and protein-DNA contacts that hold the intasome together. The N-terminal domains (NTD)s and C-terminal domains (CTD)s of the outer pair of integrase subunits are disordered and their functional roles, if any, are unclear.

Prior to the determination of the PFV intasome structure, the structures of the Tn5 transposase [12] and Mos 1 [13] transposase were the only structures of an integrase or transposase in complex with DNA and they provided the best available scaffold for modeling the active site of the HIV-1 intasome. These structures are useful for modeling the engagement of DNA with the active site of HIV-1 integrase, but are too different from retroviral integrases to model the HIV-1 intasome; the active unit is a dimer and structural similarity is limited to the catalytic domain. PFV integrase shares less than 20% sequence identity with HIV-1 integrase and has an N-terminal extension domain (NTE) that is not found in HIV-1 integrase. However, the three PFV integrase domains in the PFV intasome structure that are shared with HIV-1 integrase have essentially the same structure as the isolated HIV-1 integrase domains. Therefore, the PFV intasome structure represents a reliable scaffold onto which to model the HIV-1 intasome. The linkers between the three domains of HIV-1 integrase are shorter than in the PFV counterpart, but are sufficiently long to allow mapping of the HIV-1 integrase domains on the PFV intasome. Krishnan *et al.* [1] have used the PFV intasome structure and the partial structures of HIV-1 integrase to build a model of the HIV-1 intasome.

The model of the HIV-1 intasome provides a platform for understanding the mechanism of INSTIs such as Raltegravir and Elvitegravir. Like the structure of the PFV intasome complexed with INSTIs, inhibitor binding to the HIV-1 intasome is predicted to displace the 3′ end of the viral DNA from the active site making it unavailable as the nucleophile to attack the target DNA in the DNA strand transfer reaction. Coplanar INSTI oxygen atoms engage the divalent metal ions and the halobenzyl moieties of the INSTI stack against the penultimate cytosine of the joining strand, forcing the 3-OH of the terminal adenine to move away from the active site carboxylates and divalent metal ions. The major contact points between DNA bases and protein residues are conserved between PFV and HIV; hence, the mode of inhibitor binding is unlikely to be very different. A considerable body of data has accumulated on mutations that confer resistance to INSTIs and secondary mutations that improve viral fitness in the presence of primary resistance mutations (reviewed in [14,15]). However, only a small number of these mutations occur at residues that directly contact inhibitor in the HIV-1 intasome model. Modeling further away from the active site becomes more complicated due to divergence between the PFV and HIV-1 integrases. Clearly, actual structures of the HIV-1 intasome are required to thoroughly understand resistance mechanisms. Nevertheless, the new model is a major step forward and offers the clearest view to date on the molecular mechanism of this novel class of HIV-1 inhibitors.

## Figures and Tables

**Figure 1:**
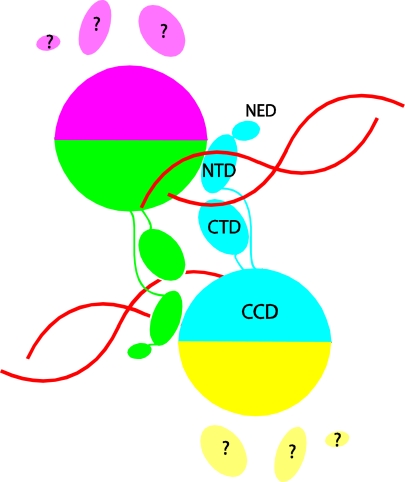
Schematic depiction of the arrangement of integrase domains in the prototype foamy virus (PFV) intasome. The four subunits in the integrase tetramer are distinguished by color. The catalytic core domain (CCD), C-terminal domain (CTD), N-terminal domain (NTD), and N-terminal extension domain (NED) of the cyan colored subunit are labeled. DNA is shown in red. The protein-protein and protein-DNA contacts that hold the tetramer together are all contributed by the inner two subunits (colored cyan and green). The CTD, NTD and NED of the outer two subunits (colored yellow and magenta) are disordered.
